# Association between organic cation transporter genetic polymorphisms and metformin response and intolerance in T2DM individuals: a systematic review and meta-analysis

**DOI:** 10.3389/fpubh.2023.1183879

**Published:** 2023-07-21

**Authors:** Aiyu Peng, Chunmei Gong, Yuanfei Xu, Xiongshun Liang, Xiaoping Chen, Wenxu Hong, Junxia Yan

**Affiliations:** ^1^Animal Laboratory, Shenzhen Center for Chronic Disease Control, Shenzhen, China; ^2^Department of Epidemiology and Health Statistics, XiangYa School of Public Health, Central South University, Changsha, China; ^3^Hunan Provincial Key Laboratory of Clinical Epidemiology, XiangYa School of Public Health, Central South University, Changsha, China; ^4^Institute of Clinical Pharmacology, Central South University, Changsha, China

**Keywords:** organic cation transporters, genetic polymorphisms, type 2 diabetes mellitus, metformin response, metformin intolerance

## Abstract

**Background:**

Variants in organic cation transporter (OCT) genes play a crucial role in metformin pharmacokinetics and are critical for diabetes treatment. However, studies investigating the effect of OCT genetic polymorphisms on metformin response have reported inconsistent results. This review and meta-analysis aimed to evaluate the associations between OCT genetic polymorphisms and metformin response and intolerance in individuals with type 2 diabetes mellitus (T2DM).

**Method:**

A systematic search was conducted on PubMed, EMBASE, CNKI, WANFANG DATA, and VIP database for identifying potential studies up to 10 November 2022. The Q-Genie tool was used to evaluate the quality of included studies. Pooled odds ratios (OR) or standardized mean differences (SMD) and 95% confidence intervals (95% CI) were calculated to determine the associations between OCT genetic polymorphisms and metformin response and intolerance that were reflected by glycemic response indexes, such as glycated hemoglobin level (HbA1c%) or change in glycated hemoglobin level (ΔHbA1c%), fasting plasma level (FPG) or change in fasting plasma glucose level (ΔFPG), the effectiveness rate of metformin treatment, and the rate of metformin intolerance. A qualitative review was performed for the variants identified just in one study and those that could not undergo pooling analysis.

**Results:**

A total of 30 related eligible studies about OCT genes (*SLC22A1, SLC22A2*, and *SLC22A3*) and metformin pharmacogenetics were identified, and 14, 3, and 6 single nucleotide polymorphisms (SNPs) in *SLC22A1, SLC22A2*, and *SLC22A3*, respectively, were investigated. Meta-analysis showed that the *SLC22A1* rs622342 polymorphism was associated with a reduction in HbA1c level (AA vs. AC: SMD [95% CI] = −0.45 [−0.73–−0.18]; *p* = 0.001). The GG genotype of the *SLC22A1* rs628031 polymorphism was associated with a reduction in FPG level (GG vs. AA: SMD [95 %CI] = −0.60 [−1.04–0.16], *p* = 0.007; GG vs. AG: −0.45 [−0.67–0.20], *p* < 0.001). No statistical association was found between the remaining variants and metformin response and intolerance.

**Conclusion:**

*SLC22A1* rs622342 and rs628031 polymorphisms were potentially associated with glycemic response to metformin. This evidence may provide novel insight into gene-oriented personalized medicine for diabetes.

## 1. Introduction

Diabetes mellitus (DM) is a global prevalent chronic metabolic disease characterized by hyperglycemia (1). The prevalence of DM among people aged 20 to 70 is approximately 10.5%, meaning one in ten adults has diabetes. DM and its complications pose a significant burden on mortality and disability globally. According to the International Diabetes Federation (IDF), there will be an estimated 784 million people with diabetes by 2045, and approximately 90% of them will have type 2 diabetes mellitus (T2DM) (2). Given its global influence, it is essential to implement measures to prevent the occurrence and development of T2DM.

Lifestyle modifications, including weight loss, increased physical activity, and healthy eating, and medication are important interventions in T2DM management. Common medications for T2DM include biguanide, sulfonylureas, thiazolidinediones, glycerides, and α-glucosidase inhibitors. Metformin is the most commonly prescribed biguanide and is considered as first-line antidiabetic drug due to its low cost, hypoglycemic effect, and few adverse reactions. Its primary mechanism seems to be a decrease in blood glucose and an improvement of insulin resistance through the inhibition of gluconeogenesis. However, there are significant inter-individual differences in metformin hypoglycemic efficacy, with more than 30% of patients not reaching the target blood glucose level after treatment (3). Metformin intolerance is mainly manifested by gastrointestinal symptoms; studies have shown that approximately 30% of patients had significant gastrointestinal intolerance when taking normal doses of metformin (4). The metformin response and intolerance are affected by non-genetic factors, such as age, sex, and physiological status, while genetic factors also play a crucial role in metformin bioavailability (5). Organic cation transporters (OCTs), belonging to the *SLC22* gene family, play an essential role in the pharmacokinetics of metformin. OCTs include three subtypes: OCT1, OCT2, and OCT3. These transporters widely distribute in human intestines, liver, kidney, and other organs and are responsible for metformin absorption, distribution, and elimination (6). Pharmacogenetic researchers speculated that genetic variants may alter the structure and function of organic cation transporters (OCTs), leading to inter-individual differences in responses to metformin (7). To date, a few studies have investigated the effect of polymorphisms in the OCT genes (*SLC22A1, SLC22A2*, and *SLC22A3*) on metformin response and intolerance. The current findings on the impact of genetic variants of these transporters on metformin clinical response and intolerance lack consistency, likely due to various factors, such as small sample sizes, differences in study groups, or observational endpoints. Thus, comprehensive meta-analyses are needed to characterize the role of genetic variants in these transporters on metformin clinical response and intolerance.

Previously, Dujic et al. (8) conducted a large-scale meta-analysis across the cohorts of the Metformin Genetics Consortium (MetGen) to investigate the effect of candidate variants of *SLC22A1* and *SLC22A2* on glycemic response to metformin. However, this meta-analysis only included patients with European ancestry who had T2DM. In recent years, a number of studies have been subsequently performed. Considering that the previous meta-analysis only included patients with European backgrounds and focused on the role of genetic variants in OCT genes on metformin response, we conducted this review and meta-analysis to estimate the association between OCT genetic polymorphisms and metformin response and intolerance in all ethnicities. This study aimed to provide more evidence and guidance for gene-oriented personalized medicine for T2DM patients.

## 2. Methods

### 2.1. Literature search strategy

Systematic searches were conducted on PubMed, Embase, China National Knowledge Infrastructure (CNKI), Wanfang database, and VIP database to retrieve potential articles up to 10 November 2022. The search strategy followed the Preferred Reporting Items for Systematic Reviews and Meta-Analyses (PRISMA) statement, and it was registered in PROSPERO (https://www.crd.york.ac.uk/PROSPERO), registration number: CRD42022326203). The search strategy was conducted by the following keywords and MeSH terms: (((((((((((((((Diabetes Mellitus, Type 2 [MeSH Terms]) OR (Diabetes Mellitus, Lipoatrophic [MeSH Terms])) OR (Non-insulin Dependent Diabetes Mellitus)) OR (Ketosis Resistant Diabetes Mellitus)) OR (Type II Diabetes Mellitus)) OR (Type 2 Diabetes Mellitus)) OR (Type 2 Diabetes)) OR (Maturity Onset Diabetes)) OR (Adult Onset Diabetes Mellitus)) OR (Stable Diabetes Mellitus)) OR (Lipoatrophic Diabetes Mellitus)) OR (NIDDM)) OR (MODY) AND (((((((((Genetic Polymorphism [MeSH Terms]) OR (Gene Polymorphism)) OR (Polymorphism)) OR (Genetic)) OR (Gene)) OR (Genetic markers)) OR (Single nucleotide polymorphism)) OR (variant)) OR (allele) AND (((((Solute Carrier Family 22 Organic cation transporters) OR (Organic cation transporters)) OR (SLC22)) OR (OCTs) AND ((((((Metformin [MeSH Terms]) OR (Dimethylbiguanidine)) OR (Dimethylguanylguanidine)) OR (Glucophage)) OR (Metformin Hydrochloride)) OR (Metformin HCl) NOT ((animals [MeSH Terms]) NOT (human [MeSH Terms]). In addition, a manual search was performed to identify potential articles that were not screened in the electronic search. After removing duplication, two authors independently screened the articles by reading the title and abstract to determine whether the study met the criteria for further reading the full text.

### 2.2. Selection criteria

We included genetic studies about OCT genetic polymorphisms and metformin monotherapy (without additional hypoglycemic medication) in T2DM patients. The duration of metformin monotherapy should be more than 2 months. The inclusion criteria were as follows: (1) case–control, cohort, or cross-sectional studies investigating the association between at least one OCT genetic variant and the therapeutic effect or adverse reaction of metformin in T2DM patients; (2) the data on defined genotype and clinical outcome were available to calculate the odds ratio (OR) or standardized mean difference (SMD) and 95% confidence intervals (95% CI); (3) all variants included in the meta-analysis should be assessed in more than one original article. The exclusion criteria included non-human studies, review papers, meta-analyses, conference abstracts, case reports, unpublished articles and comments, and studies with no metformin monotherapy or metformin treatment of < 2 months. If duplicated or overlapped studies were retrieved, we chose the largest study for further analysis.

### 2.3. Data extraction

The data were extracted independently by two authors with a preconceived table, and disagreements were resolved by consensus. Information about the first author, published year, study design, sites, characteristics of participants (percentage of males, age), treatment duration, therapeutic dose, genotyping, and relevant genes (SNPs) were extracted. Characteristics of genetic variants and related genetic association studies were collected including gene (SNP) with its chromosome position, variant type, minor allele frequency (MAF), *P*-value of HWE, and clinical effect. Clinical effect was reflected by indexes of metformin response and intolerance, including HbA1c%, ΔHbA1c%, FPG, ΔFPG, postprandial plasma glucose level (PPG), change in postprandial plasma glucose level (ΔPPG), fasting insulin level (FINS), change in fasting insulin level (ΔFINS), insulin resistance index (HOMA-IR), insulin sensitivity index (HOMA-IS), the rate of metformin response or glycemic control, and the rate of metformin intolerance. Metformin intolerance was defined as individuals who stopped metformin within the first 6 months of treatment due to the gastrointestinal adverse events caused by metformin.

### 2.4. Quality assessment of primary studies

Q-Genie tool was used to estimate the quality of included studies by two independent researchers. This tool was created and validated to aid in the quality assessment of published genetic association studies (9). It consists of 11 items that address the following 11 issues: rationale for the study, definition of outcome, selection of control group, technical and non-technical classification of exposure, discussion of sources of bias, appropriateness of sample size and power, description of planned statistical analysis and methods, statistical methods for controlling confounding, genetic hypothesis test, and appropriateness of conclusions supported by the results (6). Each item is scored from 1 (poor) to 7 (excellent) and then the scores from each item are added. Scores of ≤ 35 are considered low-quality studies, >35 and ≤ 45 are considered middle-quality studies, and >45 are considered high-quality studies.

### 2.5. Statistical analysis

Meta-analysis was conducted on variants reported in at least two published studies to evaluate their association with clinical effects. For dichotomous outcomes, we calculated the pooled ORs with 95% CIs to assess the degree of associations. For continuous outcomes, the pooled SMDs with 95% CI were calculated. The I^2^ test and Cochran Q test were used to assess heterogeneity. When a *P*-value was < 0.05 or I^2^ > 50%, heterogeneity between studies was considered to be significant, a qualitative review was performed in such cases and the potential source of heterogeneity was discussed. Otherwise, the fixed-effect model was adopted. Forest plots were used to present findings and statistical heterogeneity. STATA12.0 software was used for meta-analysis. The variants identified in only one study were qualitatively described.

## 3. Results

### 3.1. Characteristics of the included studies

**Figure 1:**
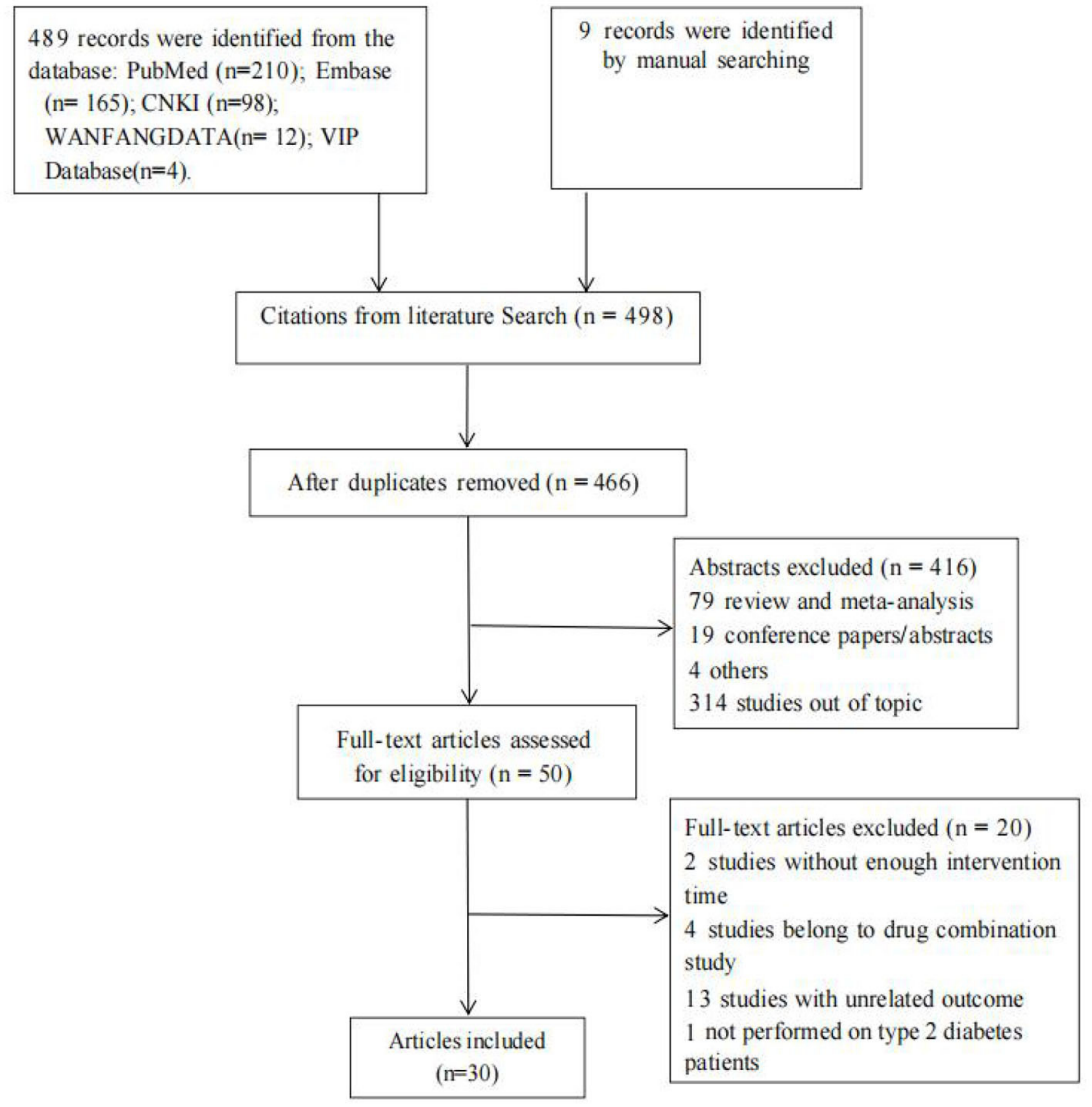
PRISMA flow diagram for the process of study selection.

A total of 495 studies were identified from PubMed (*n* = 210), Embase (*n* = 165), CNKI (*n* = 98), Wanfang Database (*n* = 12), and VIP Database (*n* = 4), and nine studies were identified by manual search (Supplementary Table 1). After screening for duplication and eligibility, 30 studies were included, and 14 studies among them were available for meta-analysis with 2,791 T2DM patients (Table 1). The flow diagram of the study selection process is shown in Figure 1.

**Table 1:** Detailed characteristics of all eligible studies.

**References**	**Year**	**Population**	**Single center/ multicenter**	**Sample size (case/ control)**	**Characteristics of the participants [%man, age (case/control)]**	**Duration of treatment**	**Dose of metformin (mg/d)**	**Genotyping**	**Gene (SNPs)**	**Q-Genie score**
**Cohort study** **(****14****)**										
Zhou et al. (10)	2009	Chinese	Single center	1,531	59%,59.1 ± 11.2/63.1 ± 10.5	≥6 months	NA	TaqMan	*SLC22A1*:rs12208357, rs72552763	51
Tkác et al. (11)	2013	Slovakian	Multicenter	148	49%,57.5 ± 0.9	6 months	1,275	real-time PCR	*SLC22A1*: rs622342 *SLC22A2*: rs316019	40
Chen (12)	2014	Chinese	Single center	82	46.3%,49.80 ± 12.18	2 months	500–1,500	XP-PCR	*SLC22A1*: rs628031 rs622342 *SLC22A2*: rs316019	40
Zhou et al. (13)	2015	Chinese	Single center	137	50.4%,56.4 ± 11.6	3 months	500–2,000	TaqMan	*SLC22A1*:rs1867351, rs4709400, rs628031, rs2297374	33
Hou et al. (14)	2015	Chinese	Single center	209	57.73%,51.9 ± 12.3(AA)/57.1 ± 7.8(Aa)/49.5 ± 13.3 (aa)	≥12 months	1,500	AS-PCR	*SLC22A2*: rs316019	34
Ghaffari-Cherati et al. (15)	2016	Iranian	Single center	150	NA,52.7 ± 10.7	3 months	1,000	PCR-RFLP	*SLC22A3*: rs3088442	32
Liu Zejing et al. (16)	2016	Chinese	Single center	105	73.3%,39.2 (35–48)	3 months	500	PCR-RFLP	LC22A1: rs628031 *SLC22A4*: rs272893	31
Fu Ting et al. (17)^*^	2016	Chinese	Single center	43	48.8%,56.25 ± 10.10	3 months	500–2,000	AS-PCR	*SLC22A1*: rs628031 rs683369 *SLC22A2*: rs316019 *SLC22A3*: rs2048327	35
Xiao et al. (18)	2016	Chinese	Single center	53	58.5%,49(29-73)	3 months	1,000–2,000	DNA DS	*SLC22A1*: rs594709	45
Hosseyni-Talei (19)	2017	Iranian	Single center	150	NA,52.7 ± 10.7	3 months	1,000	PCR-RFLP	*SLC22A3*: rs2292334	32
Mostafa-Hedeab et al. (20)	2018	Egyptian	Single center	100	NA,40.12 ± 11.46	NA	NA	TaqMan	*SLC22A1*: rs12208357	32
Reséndiz-Abarca et al. (21)	2019	Mexican	Single center	308	46.15% (25–75)	12 months	NA	TaqMan	*SLC22A1*: rs622342, rs628031,594709	44
Naja et al. (38)	2019	Lebanese	Single center	63	58.73%,54.89 ± 6.99	6 months	850	real-time PCR	*SLC22A1*: rs622342	44
Bao Xuezhi (39)	2021	Chinese	Single center	96	68.75%,51.97 ± 10.67/58.17 ± 10.82	3 months	500	PCR	*SLC22A1*: rs594709	32
**Case–Control study** **(****6****)**										
Tarasova et al. (22)	2012	Latvian	Single center	246(53/193)	30.1%,63.8 ± 8.2/58.9 ± 9.9	NA	≥500	TaqMan	*SLC22A1*: rs12208357, rs34059508, rs628031, rs72552763,rs36056065 *SLC22A2*: rs316019	50
Dujic et al. (23)	2015	Bosnia and Herzegovina	Single center	2,166 (251/1,915)	58.1%, 67.8 ± 10.5/58.0 ± 10.8	NA	1,000	TaqMan	*SLC22A1*:rs12208357 rs72552763, rs34130495, rs34059508, rs55918055	50
Dujic et al. (24)	2015	Bosnia and Herzegovina	Single center	92 (43/49)	41.3%, 57.1 ± 9.5/58.8 ± 8.4	NA	1,000	TaqMan	*SLC22A1*: rs12208357 rs72552763	44
Dawed et al. (25)	2019	IMI DIRECT cohort	Multicenter	1,414 (286/1,128)	57.5%, 60.73 ± 9.84/64.63 ± 9.91	NA	case: 1,500 (1,000–2,000) control: 1,000 (500–1,000)	PCR-RFLP	*SLC22A1*: rs12208357, rs72552763, rs34130495	54
AL-Eitan et al. (26)	2019	Jordanian	Single center	212	38.68%, 56.64 ± 9.4	≥6 months	NA	MassARRAY	*SLC22A1*: rs1867351, rs2282143, rs2282143, rs461473, rs4646272, rs622342, rs683369 *SLC22A2*: rs10755577, rs17588242,rs17589858 rs2928035, rs3127573, rs316024, rs316025, rs316026, rs533452, rs662301. *SLC22A2*: rs12194182, rs2292334, rs2504927, rs3123634	38
Marta et al. (27)	2020	Mexican	Single center	129	29.46%,53.8 ± 11.0	≥6 months	NA	TaqMan	*SLC22A1*: rs72552763 rs622342, rs34059508 rs12208357 *SLC22A2*: rs316019	50
**Nested case–control study** **(****8****)**										
Umamaheswaran et al. (28)	2015	South Indian	Single center	122 (29/93)	39%,47.79 ± 10.42/50.12 ± 9.70	3 months	500–2,250	qRT-PCR	*SLC22A1*: rs622342	33
Mahrooz et al. (29)	2015	NA	Single center	108 (59/49)	19.4%,53.16 ± 9.7	3 months	1,000	PCR-RFLP	*SLC22A1*: rs72552763	45
Fu Ting et al. (17)^*^	2016	Chinese	Single enter	130	49.23%,59.00 ± 8.14/55.01 ± 10.68	3 months	500–2,000	AS-PCR	*SLC22A1*: rs628031 rs683369 *SLC22A2*: rs316019 *SLC22A3*: rs2048327	38
Shokri et al. (30)	2016	Iranian	Single center	140 (77/63)	13.57%,52.96 ± 10.34/53.68 ± 9.68	6 months	1,000	TaqMan	*SLC22A1*: rs628031	35
Phani et al. (31)	2018	South Indian	Multicenter	188	52.13%,57.4 ± 10.6/55.8 ± 11.5	3 months	500–3,000	PCR-RFLP	*SLC22A1*: rs622342 *SLC22A2*: rs316019	42
Moeez et al. (32)	2019	Pakistani	Multicenter	600 (300/300)	48.5%,47.09 ± 12.39/46.99 ± 12.60	6 months	NA	AS-PCR	*SLC22A3*: rs3088442	33
Abrahams- October et al. (33)	2021	South African	Multicenter	140	31.43%,58.3 ± 11.4/60.7 ± 11.0	≥12 months	1,950	MassARRAY	*SLC22A1*: rs622342 rs461473 *SLC22A2*: rs316009 rs316019	36
Taheri et al. (34)	2022	Iranian	Single center	241 (98/102)	47.5%,56.99 ± 4.85/55.84 ± 4.8	6 months	1,000	ARMS- PCR	*SLC22A3*: rs543159 rs1317652	40
**Cross-sectional study** **(****3****)**										
Koshy et al. (35)	2013	South Indian	Single center	60	NA,35–55	NA	500	TaqMan	*SLC22A1*: rs12208357	29
Kashi et al. (36)	2015	Iranian	Single center	40	32.5%,52.35 ± 11.86	NA	1,000–1,500	PCR-RFLP	*SLC22A2*: rs145450955	31
Wu et al. (37)	2020	Chinese	Single center	101	60.4%,58.32 ± 10.89/57.07 ± 9.32	≥6 months	1,000–2,500	Sanger Sequencing	*SLC22A1*: rs622342	41

^*^The investigation conducted by Fu (17) consisted of two parts: One is a cohort design about metformin hypoglycemic effect and the other is a nested case–control design about metformin intolerance.

Of these studies, 14 studies were cohort studies (10–21), six were case–control studies (22–27), eight were nested case–control studies (17, 28–34), and three were cross-sectional studies (35–37). The investigation conducted by Fu (17) consisted of two parts, one is a cohort design about metformin hypoglycemic effect, and the other is a nested case–control design about metformin intolerance. Thus, we divided this investigation into two different studies in the following qualitative description and meta-analysis. Characteristics of individual studies were described in Table 1.

A total of 14, 3, and 6 single nucleotide polymorphisms (SNPs) in *SLC22A1, SLC22A2*, and *SLC22A3*, respectively, were investigated for their association with metformin response and intolerance. These studies were mostly conducted in Caucasian and Asian populations. The Q-Genie tool indicated that 5 studies were of high quality, 13 were of moderate quality, and 13 were of low quality (Supplementary Table 3). The details of genetic variants and relevant genetic association studies are shown in Table 2.

**Table 2:** Characteristics of genetic variants and related genetic association study.

**Gene**	**SNP**	**Chromosome position**	**Variant type**	**Population**	**MAF:(case/ control) or population group**	(*P*)^*^with HWE	**Clinical effects**	**References**
*SLC22A1*(OCT1)	rs622342	chr6:160151834C>A	Intron variant	Chinese	0.274	-	Patients with the A/C genotype showed better response on ΔFPG (*p =* 0.008) and ΔHbA1c (*P =* 0.006) compared with the C/C genotype. No effect on metformin intolerance	Chen (12)
				South Indian	0.205/0.245	>0.05	Significant association with the metformin response [dominant:3.85 (1.61–9.19), *P =* 0.003)][recessive:3.56 (0.83–15.26), *P =* 0.09][over-dominant:0.35 (0.14–0.86), *P =* 0.03]	Umamaheswaran et al. (28)
				Mexican	0.38	>0.05	Significant association with ΔHbA1c (*P < * 0.001)	Reséndiz-Abarca et al. (21)
				Lebanese	0.175	>0.05	Significant relationship with ΔHbA1c after 6 months of metformin treatment (*P =* 0.03) significant relationship with ΔFBS after 3 months (*P =* 0.02) and 6 months (*P =* 0.001) of metformin treatment	Naja et al. (38)
				Chinese	-	0.767	Patients with the A/A genotype were significantly higher in FPG (*P =* 0.014), HbA1c (*P =* 0.046), and HOMA-IR (*P =* 0.004).	Wu et al. (37)
				Mexican	0.358	0.188	The interaction between rs72552763 and rs622342 was associated with the metformin response (*P =* 0.024)	Marta et al. (27)
				European	0.050	0.95	No significant relationship with HbA1c decrease (*P =* 0.95)	Tkáč et al. (11)
				South Indian	0.463	-	No significant association with the metformin response (*P =* 0.88)	Phani et al. (31)
				Jordanian	0.23	0.04	No significant relationship between glycemic control (*P =* 0.432) and HbA1c level (*P =* 0.277)	AL-Eitan et al. (26)
				South African	0.259	0.218	No significant relationship with the metformin response	Abrahams-October (33)
	rs12208357	chr6:160122116C>T	Missense variant	South Indian	0.89	-	Patients with the C allele have higher FPG, PPG, and FINS (*P* < 0.05)	Koshy et al. (35)
				Bosnia and Herzegovina	-	-	Significant association with the incidence of gastrointestinal intolerance in haplotype analysis (*P =* 0.034)	Dujic et al. (24)
				Bosnia and Herzegovina	-	-	Significant association with the incidence of metformin intolerance in haplotype analysis (*P < * 0.001)	Dujic et al. (23)
				Egyptian	0.75/0.275	-	Patients with the C/G genotype showed lower RBS (*P =* 0.004) compared to patients with the C/C allele.	Mostafa-Hedeab et al. (20)
				Chinese	0.067	>0.05	No significant association with ΔHbA1c (*P =* 0.470)	Zhou et al. (10)
				Latvian	0.10	0.447	No significant effect in metformin intolerance	Tarasova et al. (22)
				European	0.071	>0.05	No significant influence on metformin intolerance	Dawed et al. (25)
	rs72552763	chr6:160139849-160139853delGAT	Deletion variant	Bosnia and Herzegovina	-	-	Significant association with the incidence of gastrointestinal intolerance in haplotype analysis (*P =* 0.034)	Dujic et al. (24)
				Bosnia and Herzegovina	-	-	Significant association with the incidence of metformin intolerance in haplotype analysis (*P < * 0.001)	Dujic et al. (23)
				Mexican	0.240	< 0.001	The interaction between rs72552763 and rs622342 was associated with metformin response (*P =* 0.024)	Marta et al. (27)
				Chinese	0.198	>0.05	No significant association with HbA1c decrease (*P =* 0.919)	Zhou et al. (10)
				Latvian	0.18	1	No significant effect in metformin intolerance	Tarasova et al. (22)
				-	0.188/0.288	0.088	No significant association with the metformin response (*P =* 0.069)	Mahrooz et al. (29)
				European	0.186	>0.05	No significant influence on metformin intolerance	Dawed et al. (25)
	rs628031	chr6:160139813A>G	Missense variant	Latvian	0.39	0.785	The A allele was significantly associated with the decrease of metformin intolerance (*P =* 0.012)	Tarasova et al. (22)
				Chinese	0.207	-	Patients with the G/G genotype showed worse response on ΔFPG (*P =* 0.019)	Chen (12)
				Chinese	0.10/0.262	0.49	Patients with the G/G genotype have shown greater reductions in the FPG level (*P < * 0.01)	Zhou et al. (10)
				Chinese	0.463/0.306	0.88	Patients with the G/G genotype have shown greater reductions in the FPG level (*P =* 0.001) The A allele was significantly associated with the increase in metformin intolerance (*P < * 0.05)	Fu (17)
				Chinese	0.325/0.307	>0.05	Patients with A/G (*P*1 = 0.038, *P*2 = 0.007) and G/G (*P*1 = 0.011, *P*2 = 0.022) genotypes showed better response on ΔFPG (1) and ΔHbA1c (2)	Liu et al. (16)
				Mexican	0.275	0.046	Significant association with ΔHbA1c (*P =* 0.016)	Reséndiz-Abarca et al. (21)
				Iranian	0.317/0.331	-	No significant effect in metformin response (*P =* 0.47)	Shokri et al. (30)
	rs594709	chr6:160134722 G>A	Intron variant	Chinese	0.268/0.286	>0.05	No significant association with ΔFPG (*P =* 0.112), ΔPPG (*P =* 0.171), and ΔHbA1c (*P =* 0.227)	Xiao et al. (18)
				Mexican	0.18	>0.05	Significant association with ΔHbA1c (*P =* 0.032)	Reséndiz-Abarca et al. (21)
				Chinese	0.268/0.29	>0.05	No significant association with Δ FPG (*P =* 0.835), ΔPPG (*P =* 0.520), and ΔHbA1c (*P =* 0.977)	Bao (39)
	rs1867351	chr6:160122091T>C	Missense variant	Chinese	0.50/0.38	0.44/0.53	Patients with the T/T genotype have shown greater reductions in PPG (*P =* 0.06) and HbA1c (*P =* 0.02) levels	Zhou et al. (13)
				Jordanian	0.19	0.85	No significant relationship with glycemic control (*P =* 0.187) and HbA1c level (*P =* 0.136)	AL-Eitan et al. (26)
	rs2297374	chr6:160154953C>T	Intron variant	Chinese	0.40/0.343	0.53/0.43	Patients with the C/T genotype have shown greater reductions in FPG (*P =* 0.002) and HbA1c (*p* =0.039) levels	Zhou et al. (13)
				Jordanian	0.46	0.79	No significant relationship with glycemic control (*P =* 0.285) and HbA1c level (*P =* 0.180)	AL-Eitan et al. (26)
	rs683369	chr6:160130172G>C	Missense variant	Chinese	0.138/0.194	0.11	No significant association with change of FPG, PPG, HbA1c	Fu (17)
				Jordanian	0.13	0.37	No significant relationship with glycemic control (*P =* 0.146) and HbA1c level (*P =* 0.072)	AL-Eitan et al. (26)
	rs34059508	chr6:160154805G>A	Missense variant	Bosnia and Herzegovina	-	-	Significant association with the incidence of metformin intolerance in haplotype analysis (*P < * 0.001)	Dujic et al. (23)
				Latvian	0.04	1	No significant effect in metformin intolerance	Tarasova et al. (22)
	rs34130495	chr6:160139792 G>A	Missense variant	Bosnia and Herzegovina	-	-	Significant association with the incidence of metformin intolerance in haplotype analysis (*P < * 0.001)	Dujic et al. (23)
				-	0.031	>0.05	No significant influence on metformin intolerance	Dawed et al. (25)
	rs461473	chr6:160122530G>A	Intron variant	Jordanian	0.10	0.44	No significant relationship with glycemic control (*P =* 0.311) and HbA1c level (*P =* 0.253)	AL-Eitan et al. (26)
				South African	0.011	0.898	No significant relationship with the metformin response	Abrahams-October et al. (33)
	rs4709400	chr6:160122578C>G	Intron variant	Chinese	0.30/0.468	0.45/0.88	Patients with the G/G genotype have shown greater reductions in FPG (*P =* 0.046) and PPG (*P =* 0.07) levels	Zhou et al. (13)
	rs36056065	G160560908delinsGT AAGTTG	Insertion variant	Latvian	0.39	0.686	Significant association with metformin intolerance (*P =* 0.002)	Tarasova et al. (22)
	rs55918055	chr6:160122197T>C	Missense variant	Scotland	-	-	Significant association with the incidence of metformin intolerance in haplotype analysis (*P < * 0.001)	Dujic et al. (23)
*SLC22A2*(OCT2)	rs316019	chr6:160249250A>C	Missense variant	Chinese	0.22	-	Patients with the C/C genotype showed better response on ΔFINS (*P =* 0.034) compared to patients with the A/C allele	Chen (12)
				South Indian	0.112	-	Significant association with the metformin response [dominant:0.35 (0.16-0.77), *P =* 0.0064)]	Phani et al. (31)
				Chinese	0.153	-	Significant association with ΔHbA1c (*P =* 0.04)	Hou et al. (14)
				European	0.065	-	No significant influence on ΔHbA1c (*P =* 0.15)	Tkáč et al. (11)
				Chinese	0.075/0.128	0.5	No significant influence on metformin intolerance (*P =* 0.445)	Fu (17)
				Latvian	0.08	0.203	No significant influence on metformin intolerance	Tarasova et al. (22)
				Mexican	0.047	*P >* 0.05	No significant influence on ΔHbA1c (*P =* 0.368)	Reséndiz-Abarca et al. (21)
	rs316009	chr6:160254732T>C	Intron variant	South African	0.039	0.595	Patients with the allele T show a better response for metformin (*P =* 0.044), but after Bonferroni correction, *P =* 0.088	Abrahams-October et al. (33)
	rs145450955	chr6:160250619C>T	Missense variant	Iranian	-	-	Patients with minor alleles had higher HbA1c level (*P =* 0.019), FPG (*P =* 0.023), and HOMA-IR (*P =* 0.03)	Kashi et al. (36)
*SLC22A3*(OCT3)	rs3088442	chr6:160451620 G>A	Non-coding transcript variant	Iranian	0.31	*P >* 0.05	No significant association between ΔHbA1c and ΔFPG	Ghaffari-Cherati et al. (15)
				Pakistani	0.153	*P >* 0.05	The allele A may act as a protective allele for metformin response [0.56 (0.40–0.80), *P < * 0.05]	Moeez et al. (32)
	rs2292334	chr6:160437156G>A	Synonymous variant	Iranian	0.35	0.544	The mean reduction in HbA1c levels following 3 months was higher in patients with the A allele than in those with the homozygous G allele	Hosseyni-Talei et al. (19)
	rs12194182	chr6:160413483T>C	Intron variant	Jordanian	0.09	0.29	Significant association with HbA1c levels (*P =* 0.007)	Al-Eitan et al. (26)
	rs543159	chr6:160354985C>A	Intron variant	Iranian	0.39/0.48	0.051/0.67	Significant association with the metformin response [Dominant:2.48(1.28–4.78), *P =* 0.0057]	Taheri et al. (34)
	rs1317652	chr6:160386129C>T	Intron variant	Iranian	0.38/0.49	0.03/0.83	Significant association with the metformin response [Dominant:2.49(1.32–4.70), *P =* 0.0043]	Taheri et al. (34)
	rs2048327	chr6:160442500T>C	Intron variant	Chinese	0.162/0.122	0.29	No significant influence on metformin intolerance (*P =* 0.813)	Fu (17)

### 3.2. Genetic effects of *SLC22A1* polymorphisms on metformin response and intolerance

A total of 25 studies evaluated the genetic effects of 14 polymorphisms of *SLC22A1* on metformin response and intolerance. These studies were mainly performed in nine countries: five countries in Asia, two in Europe, one in Latin America, and one in Africa. There were 11 cohort studies, 6 case–control studies, 6 nested case–control studies, and 2 cross-sectional studies, respectively. All details are presented in Table 1.

**Figure 2:**
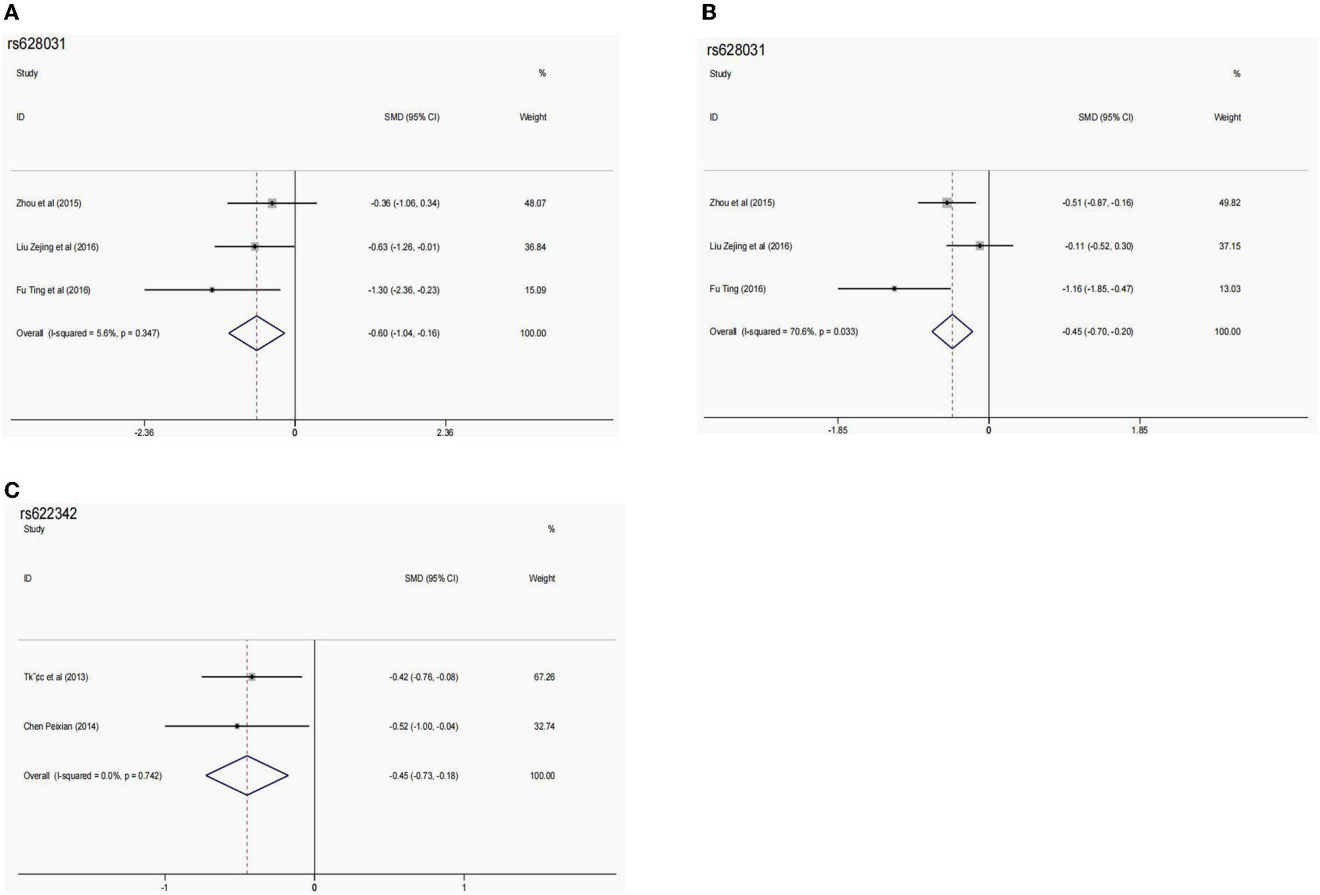
The forest plots for the association between SNPs and the metformin effect. **(A)** Forest plots of *SLC22A1* rs628031 and ΔFPG (AA vs. aa). **(B)** Forest plots of *SLC22A1* rs628031 and ΔFPG (Aa vs. aa). **(C)** Forest plots of *SLC22A1* rs622342 and ΔHbA1c% (AA vs. Aa).

rs622342 is the most commonly studied *SLC22A1* variant, and 10 studies (11, 12, 21, 26–28, 31, 33, 37, 38) assessed the association between rs622342 polymorphism and metformin responses. Six studies (12, 21, 27, 28, 37, 38) found a significant association with metformin responses in East Asian, South Asian, Middle Eastern, and Latin American populations, which was reflected by the change in HbA1c and FPG levels. While four studies (11, 26, 31, 33) found no association in South Asian, African, European, and Middle Eastern populations, one study found that rs622342 polymorphism was not associated with metformin intolerance in Chinese people. After further meta-analysis, rs622342 polymorphism was found to be substantially related to the change of HbA1c level (AA vs. AC: SMD [95% CI] = −0.45 [−0.73−0.18]; *P* = 0.001; *P*^Q^ = 0.742, I^2^ = 0.0%) (Table 3, Figure 2). But no association was found between rs622342 polymorphism and the effectiveness rate of metformin response (Supplementary Table 4).

**Table 3:** The pooled SMD (95% CIs) in meta-analysis for the association between potential SNPs and glycemic response.

**Variants**	**Study numbers**	**References**	**Sample size (AA/Aa/aa)**	**Effective marker**	**Comparison models**	**SMD**		**Model**	***P* ^Q^**	**I^2^ (%)**
						**SMD [95% CI]**	***P***			
*SLC22A1* rs628031	3	(13)	27/124/134	ΔHbA1c%	AA vs. Aa	−0.26 (−0.68–0.16)	0.229	Fixed	0.240	29.8
		(16)	27/124/134	ΔHbA1c%	Aa vs. aa	−0.45 (−0.70–−0.20)	0.102	Fixed	0.333	9.0
		(17)	27/124/134	ΔHbA1c%	AA vs. aa	−0.13 (−0.55–0.30)	0.556	Random	0.082	60.1
*SLC22A1* rs628031	3	(13)	27/124/134	ΔFPG	AA vs. Aa	−0.22 (−0.64–0.20)	0.308	Fixed	0.321	12.1
		(16)	27/124/134	ΔFPG	Aa vs. aa	−0.45 (−0.70–−0.20)	0.000	Random	0.033	70.6
		(17)	27/124/134	ΔFPG	AA vs. aa	−0.60 (−1.04–−0.16)	0.007	Fixed	0.347	5.6
*SLC22A1* rs622342	2	(11)	126/88/16	ΔHbA1c%	AA vs. Aa	−0.45 (−0.73–−0.18)	0.001	Fixed	0.742	0.0
		(12)	126/88/16	ΔHbA1c%	Aa vs. aa	0.12 (−0.42–0.67)	0.662	Fixed	0.829	0.0
			126/88/16	ΔHbA1c%	AA vs. aa	−0.32 (−0.84–−0.21)	0.241	Fixed	0.886	0.0
*SLC22A1* rs1867351	2	(26)	180/121/46	HbA1c%	AA vs. Aa	−0.18 (−0.42–0.06)	0.143	Fixed	0.642	0.0
		(13)	180/121/46	HbA1c%	Aa vs. aa	0.31 (−0.63–1.24)	0.519	Random	0.034	77.7
			180/121/46	HbA1c%	AA vs. aa	0.11 (−0.97–1.20)	0.837	Random	0.014	83.6
*SLC22A1* rs2297374	2	(26)	121/150/77	HbA1c%	AA vs. Aa	0.05 (−0.20–0.31)	0.666	Fixed	0.455	0.0
		(13)	121/150/77	HbA1c%	Aa vs. aa	−0.22 (−0.49–0.06)	0.130	Fixed	0.409	0.0
			121/150/77	HbA1c%	AA vs. aa	−0.16 (−0.47–0.16)	0.322	Fixed	0.167	47.5
*SLC22A1* rs594709	2	(18)	140/9	ΔHbA1c%	(AA+ Aa) *vs*. AA	0.16 (−0.51–0.84)	0.633	Fixed	0.528	0.0
		(39)	140/9	ΔFPG	(AA+ Aa) *vs*. AA	0.28 (−0.39–0.96)	0.416	Fixed	0.400	0.0
*SLC22A2* rs316019	3	(11, 14, 17)	317/75/8	ΔHbA1c%	AA vs. Aa	−1.15 (−2.47–0.17)	0.088	Random	0.000	94.6

A, reference allele; a, alternative allele; OR, odd ratio; HbA1c%, glycated hemoglobin level; ΔHbA1c%, change in glycated hemoglobin level; ΔFPG, change in fasting plasma glucose level; SMD, standardized mean difference; 95% CI, 95% confidence interval; *P*^Q^ value for Q-test; *P*^Z^ value for Z-test.

rs12208357 and rs72552763 polymorphisms were assessed in nine studies (10, 20, 22–25, 27, 29, 35). Three studies (20, 27, 35) demonstrated that rs12208357 and rs72552763 were significantly associated with metformin response in South Asian, African, and Latin American populations. Two studies (23, 24) reported that these two variants were associated with metformin intolerance in haplotype analysis in West European populations. Four studies (10, 22, 25, 29) showed no association in European and East Asian populations. Due to differences in study design and outcome indicators, data from related studies cannot be combined for meta-analysis.

rs628031 polymorphism was assessed by seven studies (12, 13, 16, 17, 21, 22, 30); six of them (12, 13, 16, 17, 21, 30) performed in Chinese people found significant associations between rs628031 polymorphism and metformin response, and two of them (17, 22) found that rs628031 polymorphism was significantly associated with metformin intolerance. Our meta-analysis showed that the GG genotype mediated a significantly increased change in FPG compared with AA genotype (SMD [95% CI] = −0.60 [−1.04–−0.16]; *P* = 0.007; *P*^Q^ = 0.347; I^2^ = 5.6%) and AG genotype (−0.45 [−0.70–−0.20]; *p* = 0.000; *P*^Q^ = 0.033, I^2^ = 70.6%) (Table 3, Figure 2). However, the result of the meta-analysis showed no association between this variant and metformin intolerance (Supplementary Table 4).

rs594709 polymorphism was evaluated by three studies (18, 21, 39) in East Asian and Latin American populations. The Latin American study found a significant correlation between rs594709 polymorphism and metformin hypoglycemic effect, while two studies conducted in China and our meta-analysis did not find such a correlation (Table 3). No studies have researched the relationship between rs594709 and metformin intolerance.

rs1867351, rs2297374, rs683369, rs34059508, rs34130495, and rs461473 polymorphisms were respectively assessed in two studies (13, 17, 22, 23, 25, 26, 33) in East Asian, European, and South African populations. rs1867351 and rs2297374 polymorphisms were reported to be associated with metformin for reducing blood glucose, but the results were the opposite in relevant research studies (13, 26). rs34059508 (22, 23) and rs34130495 polymorphisms (23, 25) were also reported with inconsistent results on the effect of these genetic variants on metformin intolerance. Studies showed that rs683369 (17, 26) and rs461473 polymorphisms (26, 33) were unrelated to metformin response. The pooled results showed that rs1867351 and rs2297374 polymorphisms were uncorrelated with the metformin hypoglycemic effect (Table 3). Other variants were not included in the meta-analysis due to the difference in clinical endpoints or insufficiency of genotype data. The remaining *SLC22A1* polymorphisms (rs4709400, rs36056065, and rs55918055) were assessed only in a single study in East Asian and European populations. The rs4709400 (13) polymorphism was reported to be related to hypoglycemic response and rs36056065 (22) and rs55918055 polymorphisms (23) were associated with metformin intolerance.

### 3.3. Genetic effects of *SLC22A2* polymorphisms on metformin response and intolerance

Ten studies assessed the effect of *SLC22A2* polymorphisms (rs316019, rs316009, and rs145450955) on metformin response and intolerance in six countries involving Asian and Caucasian populations.

The rs316019 polymorphism is the most studied *SLC22A2* variant, and seven studies (11, 12, 14, 17, 21, 22, 31) evaluated genetic association with metformin response or intolerance. Three studies reported that rs316019 polymorphism was associated with metformin response in East Asian (12, 14) and South Asian populations (31). The remaining studies showed that this variant was unrelated to metformin response (11, 21) and intolerance (17, 22) in East Asian, Latin American, and European populations. The heterogeneity test displayed high heterogeneity among studies (*P*^Q^ < 0.001, I^2^ = 94.6%) (Table 3).

The rs316009 polymorphism was only evaluated in African populations (33), and the study showed that the T allele has a better effect on metformin response. rs145450955 polymorphism was reported in Middle Eastern populations (36) in which patients with minor alleles exhibited worse metformin response. The results of these two variants were not pooled as they did not meet the criteria of meta-analysis.

### 3.4. Genetic effects of *SLC22A3* polymorphisms on metformin response and intolerance

Six studies (15, 17, 19, 26, 32, 34) reported the associations between *SLC22A3* polymorphisms (rs3088442, rs2292334, rs12194182, rs543159, rs1317652, and rs2048327) and metformin response or intolerance in Middle Eastern and East Asian populations.

The rs3088442 polymorphism has been investigated in two studies, which have reached opposite conclusions regarding metformin response in Middle Eastern populations (15, 32). rs2292334 (19), rs12194182 (26), rs543159 (34), and rs1317652 (34) were identified as being related to metformin response in Middle Eastern populations, respectively; in one study, rs2048327 polymorphism was reported to be unrelated to metformin intolerance in East Asian populations (17). The meta-analysis was not conducted due to the limited studies and different clinical outcomes.

## 4. Discussion

Metformin is a first-line hypoglycemic agent for T2DM patients; however, the individual metformin bioavailability is highly variable (39), which results in variation in metformin response and intolerance. The OCT genetic polymorphisms have been be considered as contributing factors to these variations. This review qualitatively and quantitatively summarized the genetic effect of *SLC22A1, SLC22A2*, and *SLC22A3* polymorphisms, respectively, on metformin response and intolerance. The pooled result showed that *SLC22A1* rs628031 and rs622342 polymorphisms were associated with hypoglycemic response to metformin, suggesting that *SLC22A1* polymorphisms play a vital role in metformin pharmacokinetics. Most of the studies were rated as low to moderate in quality due to inadequacies in identifying bias sources, sample size, power analysis, and statistical methods for controlling confounding. More high-quality studies are needed to verify these associations.

OCT1 (*SLC22A1*) is mainly expressed on the basolateral side of hepatocytes and intestinal epithelial cells, and many studies have testified that OCT1 was involved in metformin transport in the intestine and liver (40, 41). In recent years, the effect of *SLC22A1* polymorphisms on metformin treatment has been extensively studied with inconsistent outcomes. rs622342 is the most common *SLC22A1* intronic variant (42). Numerous studies have reported that the C allele mediated a diminished metformin glucose-lowering effect (27, 37, 42). The rs622342 polymorphism, although not changing the amino acid sequence, may affect the gene expression of OCT1 transport function. Our meta-analysis found that the AC genotype had a higher HbA1c reduction than the CC genotype with no significant heterogeneity. However, conflicting with these results, Dujic et al. suggested that rs622342 polymorphism has contributed little to variability in glycemic response to metformin monotherapy in T2DM patients of European ancestry (8). The discrepancy in these findings may be due to the differences in study ethnicity, sample size, and the definition of glycemic response, leading to different conclusions. rs628031 is a frequent missense variant that changes methionine to valine at position 408 (Met408Val) in the OCT1 functional protein (42), which was reported to be associated with decreased OCT1 mRNA expression in enterocytes and subsequently decreased intestinal metformin absorption and plasma concentration (43). rs628031 variant was reported with different effects on metformin response across various populations (12, 16, 17, 21, 30, 42, 44). Our meta-analysis specifically focusing on the Chinese population showed that variant homozygous exhibited greater reductions in FPG level compared with wild homozygous and variant heterozygous after metformin monotherapy. However, as only three primary studies were included in the meta-analysis, with small sample size and considerable heterogeneity, this conclusion should be cautiously drawn. There are still many *SLC22A1* polymorphisms (e.g., rs12208357, rs72552763, and rs34130495) reported to be associated with metformin response in small studies (13, 16–18, 26, 39). However, the meta-analysis of the MetGen cohort showed no significant association between *SLC22A1* variants (rs12208357 and rs72552763) and glycemic response to metformin monotherapy in European populations (8). Given the heterogeneity from the clinic and methodology in different studies reflected by the Q-Genie tool, definite conclusions about associations of the above-mentioned variants with metformin response could not be drawn. Clinical heterogeneity can be attributed to differences in inclusion and exclusion criteria, ethnicity, and intervention, such as therapeutic dose, treatment course, dosage form, and patient compliance. Methodology heterogeneity is mainly from study design, sample size, and genotyping technology. Additionally, the result of the heterogeneity test may be imprecise and biased when there were few studies included in the meta-analysis (45). Morphine is another good substrate of OCT1, and gene variants related to morphine have also been widely studied (46, 47)_._ More research studies are required to clarify the roles of pharmacogenomic variants on metformin in certain ethnic groups.

Gastrointestinal metformin intolerance is the most frequent side effect associated with metformin usage, and several studies have used its occurrence to evaluate metformin safety. OCT1 is partly responsible for metformin absorption from the intestinal lumen, and it has been suggested that reduced OCT1 could increase intestine local metformin concentration, leading to gastrointestinal intolerance (48). Common genetic polymorphisms associated with reduced-function of OCT1 include rs12208357, rs72552763, rs34130495, and rs3405950 (49). Several population observational studies (22–25) have explored the association between these polymorphisms and metformin intolerance in European populations, but the results obtained are not always consistent. However, due to limited studies, we were unable to perform a meta-analysis on these polymorphisms. In addition, we observed significant heterogeneity among these studies regarding sample size, definition of metformin intolerance, and analytical methods. Furthermore, larger multicenter studies are needed to clarify the associations between OCT1 polymorphisms and metformin intolerance.

OCT2 (*SLC22A2*), another isoform of the OCT family, is highly expressed at the basolateral membrane of renal tubule epithelium and is mainly engaged in metformin excretion in the kidney (50). A number of *SLC22A2* variants that can alter OCT2 transport function and affect glycemic response to metformin have been screened out in different populations (51). rs316019 is the most studied *SLC22A2* polymorphism. According to the pharmacokinetics study (14), the mutant allele of rs316019 may enhance the metformin glucose-lowering effect by delaying elimination. In this review, seven studies (11, 12, 14, 17, 21, 22, 31) evaluated the effect of rs316019 on metformin response or intolerance. However, our meta-analysis suggested that the rs316019 variant is not related to metformin response with significant population heterogeneity, which is in keeping with the result of the MetGen cohort performed in European populations (8). Moreover, the association between rs316019 polymorphisms and cisplatin-induced nephrotoxicity was reported extensively in clinical studies on genotype-guided prescribing (52–54). rs316009 and rs145450955 polymorphisms were reported in one study, respectively, in African (33) and Middle Eastern populations (36), and further genetic association studies are needed in the future.

OCT3 (*SLC22A3*), distributed widely in the intestine, muscle, and adipose tissue, also has a certain effect on metformin transport (55). A pharmacologic study (56) showed that *SLC22A3* variants were correlated with reduced uptake activity of metformin. However, *SLC22A3* variants have only been reported in a few population studies so far, and the result of the Q-Genie assessment suggested that the existing studies lack discussion of sources of bias and statistical methods for controlling confounding factors. Moreover, the sample size and power were not large enough to obtain statistically significant results. Therefore, it is difficult to assume an association between *SLC22A3* polymorphisms and metformin response and intolerance at present. More pharmacologic studies are required to characterize the molecular mechanism of OCT3 for transporting metformin, and well-designed multicenter RCTs should be conducted to confirm relevant findings.

There are several limitations in this review. First, due to differences in study design and outcome measures, there were limited studies accessible for meta-analysis, making it unable to further perform subgroup analysis, and meta-regression analysis to explore the source of heterogeneity. Second, variations in ethnic background and study quality were significant in studies on certain variants, so the conclusion should be cautiously drawn. Furthermore, we were unable to exclude the effect of diet and lifestyle modifications on metformin response on account of insufficient information provided by primary studies. Finally, articles written in other languages and unpublished articles with negative results were not included, which may lead to unavoidable selection bias and publication bias. Meanwhile, we were unable to check publication bias with Egger's and/or Begg–Mazumdar's test and Funnel plot due to the limited number of articles. Despite these limitations, we have summarized the effect of OCT genetic variants on metformin monotherapy as comprehensively as possible and discussed the limitations and shortcomings of current genetic association studies, which provide steering suggestions for follow-up research.

In conclusion, *SLC22A1* rs622342 and rs628031 polymorphisms were associated with metformin response, but these associations should be confirmed in more high-quality studies. Further research is also required to confirm the physiological function of OCTs and how it relates to clinical outcomes. Knowledge of the genetic effects of OCT genetic polymorphisms may provide new insights into gene-oriented personalized medicine for diabetes. With increasing international cooperation and accumulating metformin pharmacogenetic data, we hope to see uncontested data converted into clinical practice.

## Data availability statement

The original contributions presented in the study are included in the article/Supplementary material, further inquiries can be directed to the corresponding authors.

## Author contributions

JY and WH designed the entire study. YX, XL, and XC collected and analyzed the data. AP and CG wrote the manuscript. All authors read and approved the final manuscript.
