# Supplementary material for: Association between organic cation transporter genetic polymorphisms and metformin response and intolerance in T2DM individuals: a systematic review and meta-analysis
Source: Front Public Health. 2023 Jul 21;11:1183879. doi: 10.3389/fpubh.2023.1183879 (PMC10400771; doi:10.3389/fpubh.2023.1183879)
Supplement: Supplementary file 1 [file Table_1.docx]

1. Supplemental Table 1. Literature search strategy

| Pubmed |  |
| --- | --- |
| 1. (((((((((((((((((((Diabetes Mellitus, Type 2 [MeSH Terms]) OR (Diabetes Mellitus, Lipoatrophic [MeSH Terms])) OR (Non-insulin-Dependent Diabetes Mellitus [Title/Abstract])) OR (Non insulin Dependent Diabetes Mellitus [Title/Abstract])) OR (Ketosis-Resistant Diabetes Mellitus [Title/Abstract])) OR (Ketosis Resistant Diabetes Mellitus [Title/Abstract])) OR (Type II Diabetes Mellitus [Title/Abstract])) OR (Type 2 Diabetes Mellitus [Title/Abstract])) OR (Type 2 Diabetes [Title/Abstract])) OR (Maturity-Onset Diabetes [Title/Abstract])) OR (Maturity Onset Diabetes [Title/Abstract])) OR (Adult-Onset Diabetes Mellitus [Title/Abstract])) OR (Adult Onset Diabetes Mellitus [Title/Abstract])) OR (Stable Diabetes Mellitus [Title/Abstract])) OR (Lipoatrophic Diabetes Mellitus [Title/Abstract])) OR (NIDDM [Title/Abstract])) OR (MODY [Title/Abstract]) | 213453 |
| 1. (((((((((Genetic Polymorphism [MeSH Terms]) OR (Gene Polymorphism [Title/Abstract])) OR (Polymorphism [Title/Abstract])) OR (Genetic [Title/Abstract])) OR (Gene [Title/Abstract])) OR (Genetic markers [Title/Abstract])) OR (Single nucleotide polymorphism [Title/Abstract])) OR (variant [Title/Abstract])) OR (allele [Title/Abstract]) | 2755425 |
| 1. (((((Solute Carrier Family 22 Organic Cation Transporters) OR (Organic Cation Transporters)) OR (SLC22)) OR (OCT) | 3257807 |
| 1. (((((((Metformin [MeSH Terms]) OR (Dimethylbiguanidine [Title/Abstract])) OR (Dimethylguanylguanidine [Title/Abstract])) OR (Glucophage [Title/Abstract])) OR (Metformin Hydrochloride [Title/Abstract])) OR (Hydrochloride, Metformin [Title/Abstract])) OR (Metformin HCl [Title/Abstract]) | 16481 |
| 1. ((animals [Mesh Terms]) NOT (human [MeSH Terms]) | 4997089 |
| 1. 1 AND 2 AND 3 AND 4 NOT 5 | 210 |

| Embase |  |
| --- | --- |
| 1. 'diabetes mellitus, type 2'/exp OR 'diabetes mellitus, lipoatrophic'/exp OR 'non-insulin-dependent diabetes mellitus': ab, ti OR 'non insulin dependent diabetes mellitus': ab, ti OR 'ketosis-resistant diabetes mellitus': ab, ti OR 'ketosis resistant diabetes mellitus': ab, ti OR 'type ii diabetes mellitus': ab, ti OR 'type 2 diabetes mellitus': ab, ti OR 'type 2 diabetes': ab, ti OR 'maturity-onset diabetes': ab, ti OR 'maturity onset diabetes': ab, ti OR 'adult-onset diabetes mellitus': ab, ti OR 'adult onset diabetes mellitus': ab, ti OR 'stable diabetes mellitus': ab, ti OR 'lipoatrophic diabetes mellitus': ab, ti OR NIDDM: ab, ti OR MODY: ab, ti | 343824 |
| 2. 'genetic polymorphism'/exp OR 'gene polymorphism’: ab, ti OR polymorphism: ab, ti OR genetic: ab, ti OR gene: ab, ti OR 'genetic markers': ab, ti OR 'single nucleotide polymorphism': ab, ti OR variant: ab, ti OR allele: ab, ti | 3435570 |
| 3.solute AND carrier AND family AND 22 AND organic AND cation AND transporter OR (organic AND cation AND transporter) OR slc22 OR oct | 736431 |
| 4. 'metformin'/exp OR dimethylbiguanidine: ab, ti OR dimethylguanylguanidine: ab, ti OR glucophage: ab, ti OR 'metformin hydrochloride': ab, ti OR 'hydrochloride, metformin': ab, ti OR 'metformin hcl': ab, ti | 75471 |
| 5. 'animals'/exp NOT 'human'/exp | 5769386 |
| 6. 1 AND 2 AND 3 AND 4 NOT 5 | 165 |

| CNKI |  |
| --- | --- |
| 1. (TKA=Type 2 diabetes mellitus) OR (TKA=Type Ⅱdiabetes mellitus) OR (TKA=Lipoatrophic diabetes mellitus) OR (TKA=Maturity-onset diabetes) OR (TKA= Adult-onset diabetes mellitus) OR (TKA=Stable diabetes mellitus) OR (TKA=Ketosis-resistant diabetes mellitus) OR (TKA= Non-insulin-dependent diabetes mellitus) OR (TKA=MODY) OR (TKA=NIDDM) | 165277 |
| 1. (TKA=Genetic polymorphism) OR (TKA=Genetic variant) OR (TKA=Genetic markers) OR (TKA= Single nucleotide polymorphism) OR (TKA=Genetic association) OR (TKA=Gene) | 1187223 |
| 3. (TKA=Solute Carrier Family 22 Organic Cation Transporters) OR (TKA=Organic Cation Transporters) OR (TKA=SLC22) OR (TKA=OCT) | 37957 |
| 4. (TKA= Metformin) OR (TKA= Metformin Hydrochloride) OR (TKA= Glucophage) | 26923 |
| 5. 1 AND 2 AND 3 AND 4 | 98 |

| Wanfang |  |
| --- | --- |
| 1. (主题：Type 2 diabetes mellitus) OR (主题：Type Ⅱdiabetes mellitus) OR (主题：Lipoatrophic diabetes mellitus) OR (主题： Maturity-onset diabetes) OR (主题：Adult-onset diabetes mellitus) OR (主题：Stable diabetes mellitus) OR (主题：Ketosis-resistant diabetes mellitus) OR (主题：Non-insulin-dependent diabetes mellitus) OR (主题：MODY) OR (主题：NIDDM) | 226380 |
| 1. (主题：Genetic polymorphism）OR (主题：Genetic variant) OR (主题：Genetic markers) OR (主题：Single nucleotide polymorphism) OR (主题：Genetic association) OR (主题：Gene) | 1240568 |
| 3. (主题：Solute Carrier Family 22 Organic Cation Transporters) OR (主题：Organic Cation Transporters) OR (主题：SLC22) OR (主题：OCT) | 113536 |
| 1. (主题：Metformin) OR (主题：Metformin Hydrochloride) OR (主题：Glucophage) | 29104 |
| 1. 1 AND 2 AND 3 AND 4 | 12 |

| VIP database |  |
| --- | --- |
| 1. M= (Type 2 diabetes mellitus OR Type Ⅱdiabetes mellitus OR Lipoatrophic diabetes mellitus OR Maturity-onset diabetes OR Adult-onset diabetes mellitus OR Stable diabetes mellitus OR Ketosis-resistant diabetes mellitus OR Non-insulin-dependent diabetes mellitus OR MODY OR NIDDM) | 107052 |
| 1. M= (Genetic polymorphism OR Genetic variant OR Genetic markers OR Single nucleotide polymorphism OR Genetic association OR Gene) | 506239 |
| 1. M= (Solute Carrier Family 22 Organic Cation Transporters OR Organic Cation Transporters OR SLC22 OR OCT) | 3698 |
| 1. M= (Metformin OR Metformin Hydrochloride OR Glucophage) | 13306 |
| 1. 1 AND 2 AND 3 AND 4 | 4 |

2.Supplemental Table 2: Literature list for full-text reading

| No | Authors | Title | Published  Year | Journal | Included or excluded | Reasons |
| --- | --- | --- | --- | --- | --- | --- |
| 1 | Al-Eitan, L. N. Almomani, B. A. Nassar, A. M. Elsaqa, B. Z. Saadeh, N. A. | Metformin pharmacogenetics: effects of SLC22A1, SLC22A2, andSLC22A3 polymorphisms on glycemic control and HbA1c levels | 2019 | Journal of personalized Medicine | Included |  |
| 2 | Abrahams-October, Z. Xhakaza, L. Pearce, B. Mandisa, M. C. Benjeddou, M. Vincent, A. O. Johnson, R. Jebio, O. J. | Genetic association of solute carrier transporter gene variants with metformin response | 2021 | Balkan Journal of Medical Genetics | Included |  |
| 3 | Choi, J. H. Yee, S. W. Ramirez, A. H. Morrissey, K. M. Jang, G. H. Joski, P. J. Mefford, J. A.  Hesselson, S. E. Schlessinger, A. Jenkins, G. Castro, R. A.  Johns, S. J. Stryke, D. Sali, A.  Ferrin, T. E. Witte, J. S. Kwok, P. Y. Roden, D. M. Wilke, R. A.  McCarty, C. A. Davis, R. L.  Giacomini, K. M. | A common 5'-UTR variant in MATE2-K is associated with poor response to metformin | 2011 | Clinical Pharmacology & Therapeutics | Excluded | Unrelated outcomes |
| 4 | Christensen, M. M. H.  Andersen, C. B. Damkier, P.  Nielsen, H. B. Brøsen, K | Focused Conference Group: P02 - Transmembrane transport: Perspectives for disease and drug discovery pharmacogenetics of metformin | 2010 | Basic and Clinical Pharmacology and Toxicology | Excluded | Drug combination |
| 5 | Chen, L. Takizawa, M. Chen, E.  Schlessinger, A. Segenthelar, J.  Choi, J. H. Sali, A. Kubo, M.  Nakamura, S. Iwamoto, Y.  Iwasaki, N. Giacomini, K. M. | Genetic polymorphisms in organic cation transporter 1 (OCT1) in Chinese and Japanese populations exhibit altered function | 2010 | Journal of Pharmacology and Experimental Therapeutics | Excluded | Unrelated outcomes |
| 6 | Dawed, A. Y. Zhou, K.  van Leeuwen, N. Mahajan, A.  Robertson, N. Koivula, R.  Elders, P. J. M. Rauh, S. P.  Jones, A. G. Holl, R. W.  Stingl, J. C. Franks, P. W.  McCarthy, M. I. Hart, L. M.  Pearson, E. R. | Variation in the plasma membrane monoamine transporter (PMAT) (encoded by SLC29A4) and organic cation transporter 1 (OCT1) (encoded by SLC22A1) and gastrointestinal intolerance to metformin in type 2 diabetes: An IMI direct study | 2019 | Diabetes Care | Included |  |
| 7 | Dujic, T. Causevic, A. Bego, T.  Malenica, M. Velija-Asimi, Z.  Pearson, E. R. Semiz, S. | Organic cation transporter 1 variants and gastrointestinal side effects of metformin in patients with Type 2 diabetes | 2015 | Diabetic Medicine | Included |  |
| 8 | Dujic, T. Zhou, K. Donnelly, L. A. Tavendale, R. Palmer, C. N. A. Pearson, E. R. | Association of organic cation transporter 1 with intolerance to metformin in type 2 diabetes: A GoDARTS study | 2015 | Diabetes | Included |  |
| 9 | Ghaffari-Cherati, M. Mahrooz, A. Hashemi-Soteh, M. B.  Hosseyni-Talei, S. R. Alizadeh, A. Nakhaei, S. M. | Allele frequency and genotype distribution of a common variant in the 3´-untranslated region of the SLC22A3 gene in patients with type 2 diabetes: association with response to metformin | 2016 | Journal of Research in Medical Sciences | Included |  |
| 10 | Goswami, S. Yee, S. W. Xu, F.  Sridhar, S. B. Mosley, J. D.  Takahashi, A. Kubo, M.  Maeda, S. Davis, R. L.  Roden, D. M. Hedderson, M. M. Giacomini, K. M. Savic, R. M. | A longitudinal HbA1c model elucidates genes linked to disease progression on metformin | 2016 | Clinical Pharmacology & Therapeutics | Excluded | Unrelated outcomes |
| 11 | Hosseyni-Talei, S. R. Mahrooz, A. Hashemi-Soteh, M. B.  Ghaffari-Cherati, M. Alizadeh, A. | Association between the synonymous variant organic cation transporter 3 (OCT3)-1233G>A and the glycemic response following metformin therapy in patients with type 2 diabetes | 2017 | Iranian Journal of Basic Medical Sciences | Included |  |
| 12 | Hou, W. Zhang, D. Lu, W.  Zheng, T. Wan, L. Li, Q. Bao, Y.  Liu, F. Jia, W. | Polymorphism of organic cation transporter 2 improves glucose-lowering effect of metformin via influencing its pharmacokinetics in Chinese type 2 diabetic patients | 2015 | Molecular Diagnosis and Therapy | Included |  |
| 13 | Kashi, Z. Masoumi, P.  Mahrooz, A. Hashemi-Soteh, M. B. Bahar, A. Alizadeh, A. | The variant organic cation transporter 2 (OCT2)-T201M contribute to changes in insulin resistance in patients with type 2 diabetes treated with metformin | 2015 | Diabetes Research and Clinical Practice | Included |  |
| 14 | Koshy, M. Sethupathy, S. Annamalai, P.T. Renju, V.C. Santha, K. | [Association of oct1 gene polymorphism with glycemic status and serum metformin levels in type ii diabetes mellitus patients](https://www.embase.com/search/results?subaction=viewrecord&rid=1&page=1&id=L368928606) | 2013 | International Journal of Pharmaceutical Sciences and Research | Included |  |
| 15 | Klen, J. Goričar, K. Janež, A.  Dolžan, V. | The role of genetic factors and kidney and liver function in glycemic control in type 2 diabetes patients on long-term metformin and sulphonylurea cotreatmen | 2014 | BioMed Research International | Excluded | Drug combination |
| 16 | Mahrooz, A. Parsanasab, H.  Hashemi-Soteh, M. B. Kashi, Z. Bahar, A. Alizadeh, A.  Mozayeni, M. | The role of clinical response to metformin in patients newly diagnosed with type 2 diabetes: a monotherapy study | 2015 | Clinical and Experimental Medicine | Included |  |
| 17 | Marta, M. Sánchez-Pozos, K.  Jaimes-Santoyo, J.  Monroy-Escutia, J.  Rivera-Santiago, C.de Los Ángeles Granados-Silvestre, M. Ortiz-López, M. G. | Pharmacogenetic evaluation of metformin and Sulphonylurea response in Mexican mestizos with type 2 diabetes | 2020 | Current Drug Metabolism | Included |  |
| 18 | Moeez, S. Riaz, S. Masood, N.  Kanwal, N. Arif, M. A. Niazi, R. Khalid, S. | Evaluation of the rs3088442 G>A SLC22A3 gene polymorphism and the role of microRNA 147 in groups of adult Pakistani populations with type 2 diabetes in response to metformin. | 2019 | Canadian journal of diabetes | Included |  |
| 19 | Moeez, S. Khalid, Z. Jalil, F.  Irfan, M. Ismail, M. Arif, M. A.  Niazi, R. Khalid, S. | Effects of SLC22A2 (rs201919874) and SLC47A2 (rs138244461) genetic variants on metformin pharmacokinetics in Pakistani T2DM patients | 2019 | Journal of Pakistan Medical Association | Excluded | Drug combination |
| 20 | Gomaa, MH. Alaa, A. M. Gamal, T. Dina, S. Randa, F. S. Manal, E. H. | A common 5′-UTR variant in MATE2-K is associated with poor response to metformin | 2018 | Biomedical and Pharmacology Journal | Included |  |
| 21 | Naja, K. EI Shamieh, S.  Fakhoury, R. | rs622342A>C in SLC22A1 is associated with metformin pharmacokinetics and glycemic response | 2019 | Drug Metabolism and Pharmacokinetics | Included |  |
| 22 | Ningrum, V. D. A. Ikawati, Z.  Sadewa, A. H. Ikhsan, M. R. | Allele frequencies of two main metformin transporter genes: SLC22A1 rs628031 A>G and SLC47A1 rs2289669 G>A among the javanese population in Indonesia | 2017 | Current Pharmacogenomics and Personalized Medicine | Excluded | Unrelated outcomes |
| 23 | Ningrum, V. D. A.  Istikharah, R.  Firmansyah, R. | Allele frequency of SLC22A1 Met420del metformin main transporter encoding gene among javanese-indonesian population | 2019 | Open Access Macedonian Journal of Medical Sciences | Excluded | Unrelated outcomes |
| 24 | Ortiz-Lopez, M. G.  Sanchez-Pozos, K. A.  Garcia-Rodriguez, H.  Santoyo, J. J.  Peña-Espinoza, B. I.  Granados-Silvestre, M. A.  Menjivar, M. | Evaluation of CY2C9 And OCT polymorphisms in response to therapy with metformin and sulphonylureas in patients with type 2 diabetes | 2018 | Endocrine Reviews | Excluded | Drug combination |
| 25 | Phani, N. M. Vohra, M. Kakar, A. Adhikari, P. Nagri, S. K.  D'Souza, S. C. Umakanth, S.  Satyamoorthy, K. Rai, P. S. | Implication of critical pharmacokinetic gene variants on therapeutic response to metformin in Type 2 diabetes | 2018 | Pharmacogenomics | Included |  |
| 26 | Reséndiz-Abarca, C. A.  Flores-Alfaro, E.  Suárez-Sánchez, F.  Cruz, M. Valladares-Salgado, A. Del Carmen Alarcón-Romero, L. Vázquez-Moreno, M. A.  Wacher-Rodarte, N. A.  Gómez-Zamudio, J. H. | Altered glycemic control associated with polymorphisms in the SLC22A1 (OCT1) gene in a Mexican population with type 2 diabetes mllitus treated with metformin: a cohort study | 2019 | The Journal of Clinical Pharmacology | Included |  |
| 27 | Shibata, Yamamoto, R.  Takano, Stigmas, C.  Ikeda, Tostado, Kediri, I. | Human organic cation transporter (OCT1 and OCT2) gene polymorphisms and therapeutic effects of metformin | 2007 | Journal of Human Genetics | Excluded | Unrelated outcomes |
| 28 | Shu, Y. Sheardown, S. A. Brown, C. Owen, R. P. Zhang, S. Castro, R. A. Ianculescu, A. G. Yue, L. Lo, J. C. Burchard, E. G. Brett, C. M. & Giacomini, K. M. | Effect of genetic variation in the organic cation transporter 1 (OCT1) on metformin action | 2007 | Journal of Clinical Investigation | Excluded | Not performed on type 2 diabetes patients |
| 29 | Shu, Y. Brown, C. Castro, R. A. Shi, R. J. Lin, E. T., Owen, R. P. Sheardown, S. A. Yue, L. Burchard, E. G. Brett, C. M. & Giacomini, K. M. | Effect of genetic variation in the organic cation transporter 1, OCT1, on metformin pharmacokinetics | 2008 | Clinical Pharmacology and Therapeutics | Excluded | Unrelated outcomes |
| 30 | Shokri, F. Gheada, H. Ghafoor F. S. Movafagh, A. Abediankenari, S. Mahrooz, A. Kashi, Z. Omrani, M. D. | Impact of ATM and SLC22A1 polymorphisms on therapeutic response to metformin in Iranian diabetic patients | 2016 | International Journal of Clinical and Experimental Pathology | Included |  |
| 31 | Taheri, R. Kazerouni, F.  Mirfakhraei, R. Kalbasi, S.  Shahrokhi, S. Z. Rahimipour, A. | The influence of SLC22A3 rs543159 and rs1317652 genetic variants on metformin therapeutic efficacy in newly diagnosed patients with type 2 diabetes mellitus: 25 weeks follow-up study | 2022 | Gene | Included |  |
| 32 | Tarasova, L. Kalnina, I.  Bumbure, A. Geldnere, K.  Ritenberga, R. Nikitina-Zake, L. Vaivade, I. Pirags, V. Klovins, J. | Influence of genetic variations in organic cation transporter 1 and 2 and MATE1 on efficiency and safety of antidiabetic peroral drug metformin therapy | 2012 | European Journal of Pharmaceutical Sciences | Included |  |
| 33 | Tkáč, I. Klimčáková, L. Javorský, M. Fabianová, M.  Schroner, Z. Hermanová, H.  Babjaková, E. Tkáčová, R. | Pharmacogenomic association between a variant in SLC47A1 gene and therapeutic response to metformin in type 2 diabetes | 2013 | Diabetes, Obesity and Metabolism | Included |  |
| 34 | Tzvetkov, M. V. Vormfelde, S. V. Balen, D. Meineke, I. Schmidt, T. Sehrt, D. Sabolić, I. Koepsell, H. Brockmöller, J. | The effects of genetic polymorphisms in the organic cation transporters OCT1, OCT2, and OCT3 on the renal clearance of metformin | 2009 | Clinical Pharmacology and Therapeutics | Excluded | Unrelated outcomes |
| 35 | Umamaheswaran, G.  Praveen, R. G.  Damodaran, S. E.  Das, A. K.  Adithan, C. | Influence of SLC22A1 rs622342 genetic polymorphism on metformin response in South Indian type 2 diabetes mellitus patients | 2015 | Clinical and Experimental Medicine | Included |  |
| 36 | Wu, K. Li, X. Xu, Y. Zhang, X.  Guan, Z. Zhang, S. Li, Y. | SLC22A1 rs622342 polymorphism predicts insulin resistance improvement in patients with type 2 diabetes mellitus treated with metformin: a cross-sectional study | 2020 | International Journal of Endocrinology | Included |  |
| 37 | Xiao, D. Guo Y, Li, X, Yin, J. Y, Zheng, W, Qiu, X. W, Xiao, Liu, R. R, Wang, S. Y, Gong, W. J, Zhou, H. H. Liu, Z. Q. | The impacts of SLC22A1 rs594709 and SLC47A1 rs2289669 polymorphisms on metformin therapeutic efficacy in Chinese  type 2 diabetes patients | 2016 | International Journal of Endocrinology | Included |  |
| 38 | Zhou, K. Donnelly, L. A.  Kimber, C. H. Donnan, P. T.  Doney, A. S. F. Leese, G.  Hattersley, A. T. McCarthy, M. I.Morris, A. D. Palmer, C. N. A. Pearson, E. R. | Reduced-function SLC22A1 polymorphisms encoding organic cation transporter 1 and glycemic response to metformin: A GoDARTS study | 2009 | Diabetes | Included |  |
| 39 | Zhou, Y. Ye, W. Wang, Y.  Jiang, Z. Meng, X. Xiao, Q.  Zhao, Q. Yan, J. | Genetic variants of OCT1 influence glycemic response to metformin in Han Chinese patients with type-2 diabetes mellitus in Shanghai | 2015 | International Journal of Clinical and Experimental Pathology | Included |  |
| 40 | Zaharenko, L. Kalnina, I.  Geldnere, K. Konrade, I.  Grinberga, S. Židzik, J.  Javorský, M. Lejnieks, A.  Nikitina-Zake, L. Fridmanis, D.  Peculis, R. Radovica-Spalvina, I. Hartmane, D. Pugovics, O.  Tká, I. Klimáková, L. Pirags, V.  Klovins, J. | Single nucleotide polymorphisms in the intergenic region between metformin transporter OCT2 and OCT3 coding genes are associated with short-Term response to metformin monotherapy in type 2 diabetes mellitus patients | 2016 | European Journal of Endocrinology | Excluded | Intervention time not enough |
| 41 | Bao, X. Z. | Study on transporter gene polymorphism and metformin individ ualized medication in Mongolian type 2 diabetes patients in Tong liao area, Inner Mongolia | 2021 | Thesis | Included |  |
| 42 | Chen, P. X. | Effect of SLC22A1, SLC22A2, SLC47A1, SLC47A2, ATM gene polymorphism on response of the efficacy and the adverse reaction of metformin in type 2 diabetes | 2014 | Thesis | Included |  |
| 43 | Chen, M. | Analysis of clinical phenotype and factors of adverse reactions of metformin | 2020 | Thesis | Excluded | Unrelated outcomes |
| 44 | Fu, T. | Correlations between genetic variations of transports with the gastrointestinal side effect and therapeutic efficacy of metformin in patients with type 2 diabetes | 2016 | Thesis | Included |  |
| 45 | Liu, Z. J. Liu, X. Y. Li, J. Wang, J. Liu, Q. | association between SLC22A1，SLC22A4 gene polymorphisms and therapeutic efficacy of metformin in type 2 diabetes mellitus | 2016 | Journal of Clinical Laboratory Science | Included |  |
| 46 | Mayila, A. Z. Z. Wang, N. Zhao, J. Yu, X. M. He, W. F. | SLC22A2 gene polymorphism in Xinjiang Uygur population | 2019 | China Medicine | Excluded | Unrelated outcomes |
| 47 | Wang, T. | Short-term intervention of metformin in Xinjiang Uygur patients with type 2 diabetes or IFG; the influence of OCT1 gene polymorphisms on the metformin response in patients with type 2 diabetes or impaired glucose metabolism | 2008 | Thesis | Excluded | Intervention time not enough |
| 48 | Zhang, J. J. | Polymorphism distribution of SLC22A1 rs12208357 in healthy Uygur population of Xinjiang | 2018 | Thesis | Excluded | Unrelated outcomes |
| 49 | Zhang, L. | Frequency distribution of SLC22A1gene  polymorphism in Uygur young men in Xinjiang | 2017 | Thesis | Excluded | Unrelated outcomes |
| 50 | Zhu, L. | The relationship between SLC22A1 and Cllorf65 gene polymorphism and the efficacy of metformin in patients with type 2 diabetes | 2021 | Systems Medicine | Excluded | Unrelated outcomes |

1. Supplemental Table 2: Quality of genetic association studies (Q-Genie)

| First Author(year) | Item 1 | Item 2 | Item 3 | Item 4 | Item 5 | Item 6 | Item 7 | Item 8 | Item 9 | Item 10 | Item 11 | Score | Levels |
| --- | --- | --- | --- | --- | --- | --- | --- | --- | --- | --- | --- | --- | --- |
| Zhou et al (2015) | 2 | 3 | 5 | 3 | 3 | 3 | 2 | 4 | 3 | 4 | 1 | 33 | 1 |
| Tarasov et al (2012) | 4 | 4 | 5 | 5 | 3 | 5 | 5 | 5 | 5 | 4 | 5 | 50 | 3 |
| Shokri et al (2016) | 4 | 4 | 5 | 4 | 3 | 1 | 1 | 3 | 2 | 4 | 4 | 35 | 1 |
| Reséndiz-Abarca et al (2019) | 4 | 4 | 5 | 3 | 3 | 4 | 5 | 3 | 4 | 5 | 4 | 44 | 2 |
| Zhou et al (2009) | 5 | 5 | 5 | 4 | 3 | 5 | 5 | 5 | 5 | 4 | 5 | 51 | 3 |
| Mostafa-Hedeab et al (2018) | 4 | 2 | 4 | 3 | 3 | 1 | 5 | 2 | 1 | 3 | 4 | 32 | 1 |
| Koshy et al (2013) | 3 | 4 | 3 | 4 | 3 | 1 | 5 | 2 | 1 | 2 | 1 | 29 | 1 |
| Mahrooz et al (2015) | 4 | 5 | 5 | 4 | 3 | 5 | 1 | 5 | 4 | 4 | 5 | 45 | 2 |
| Dawed et al (2019) | 5 | 4 | 5 | 6 | 4 | 5 | 5 | 6 | 5 | 4 | 5 | 54 | 3 |
| Xiao et al (2016) | 5 | 4 | 4 | 3 | 3 | 5 | 3 | 4 | 5 | 4 | 5 | 45 | 2 |
| Wu et al (2020) | 4 | 4 | 4 | 4 | 3 | 3 | 3 | 5 | 4 | 3 | 4 | 41 | 2 |
| Umamaheswaran et al (2015) | 5 | 3 | 3 | 4 | 3 | 1 | 3 | 3 | 1 | 4 | 3 | 33 | 1 |
| Tkác et al (2013) | 5 | 4 | 5 | 2 | 3 | 4 | 4 | 3 | 5 | 3 | 3 | 41 | 2 |
| Naja et al (2019) | 4 | 4 | 5 | 3 | 3 | 4 | 5 | 4 | 4 | 4 | 4 | 44 | 2 |
| Marta et al (2020) | 5 | 4 | 4 | 6 | 5 | 4 | 3 | 6 | 4 | 4 | 5 | 50 | 3 |
| AL-Eitan et al (2019) | 4 | 4 | 4 | 4 | 3 | 3 | 2 | 4 | 3 | 3 | 4 | 38 | 2 |
| Liu Zejing（2016） | 3 | 4 | 4 | 3 | 3 | 1 | 3 | 3 | 1 | 3 | 3 | 31 | 1 |
| Fu Ting (2016) cohort study | 3 | 4 | 4 | 4 | 3 | 4 | 2 | 4 | 1 | 3 | 3 | 35 | 1 |
| Fu Ting (2016) case-control study | 3 | 4 | 4 | 4 | 3 | 4 | 1 | 4 | 4 | 4 | 3 | 38 | 2 |
| Chen Peixian (2014) | 5 | 3 | 4 | 4 | 3 | 5 | 1 | 4 | 3 | 3 | 5 | 40 | 2 |
| Bao Xuezhi (2021) | 3 | 3 | 4 | 3 | 3 | 2 | 1 | 4 | 3 | 3 | 3 | 32 | 1 |
| Phani et al (2018) | 4 | 4 | 4 | 4 | 3 | 5 | 3 | 5 | 3 | 4 | 3 | 42 | 2 |
| Abrahams-October et al (2021) | 5 | 3 | 4 | 2 | 3 | 3 | 1 | 3 | 4 | 3 | 5 | 36 | 2 |
| Hou et al (2015) | 4 | 4 | 4 | 4 | 3 | 2 | 2 | 3 | 1 | 3 | 4 | 34 | 1 |
| Kashi et al (2015) | 3 | 4 | 4 | 3 | 3 | 3 | 1 | 3 | 1 | 2 | 4 | 31 | 1 |
| Moeez et al (2019) | 4 | 3 | 3 | 2 | 3 | 3 | 5 | 4 | 1 | 3 | 2 | 33 | 1 |
| Ghaffari‑Cherati et al (2016) | 4 | 4 | 4 | 3 | 3 | 3 | 1 | 2 | 1 | 3 | 4 | 32 | 1 |
| Hosseyni-Talei et al (2017) | 4 | 4 | 4 | 3 | 3 | 3 | 1 | 2 | 1 | 3 | 4 | 32 | 1 |
| Taheri et al (2022) | 4 | 4 | 4 | 3 | 3 | 4 | 3 | 4 | 3 | 4 | 4 | 40 | 2 |
| Dujic et al (2015) (Published in November) | 4 | 4 | 4 | 4 | 4 | 4 | 5 | 3 | 4 | 4 | 4 | 44 | 2 |
| Dujic et al (2015) (published in May) | 4 | 4 | 4 | 4 | 4 | 5 | 6 | 5 | 5 | 4 | 5 | 50 | 3 |

1. Supplemental Table 4: The pooled OR (95% CIs) in meta-analysis for the association between potential SNPs and therapeutic response

| Variants | Study  numbers | Study | Sample size  (case/control) | Effective marker | Comparison models | OR | | Model | *P*^Q^ | I^2^(%) |
| --- | --- | --- | --- | --- | --- | --- | --- | --- | --- | --- |
|  |  |  |  |  |  | OR [95% CI] | *P* |  |  |  |
| *SLC22A1* rs628031 | 2 | Tarasova et al(22) | 91/278 | Metformin intolerance | Dominant model | 1.29 (0.17-9.49) | 0.804 | Random | 0.018 | 82.2 |
|  |  | Fu Ting et al(17) | 91/278 | Metformin intolerance | Recessive model | 1.21 (0.21-6.94) | 0.832 | Random | 0.001 | 90.9 |
|  |  |  | 91/278 | Metformin intolerance | Additive model | 0.91 (0.16-5.08) | 0.910 | Random | 0.000 | 92.4 |
| *SLC22A1* rs622342 | 3 | Umamaheswaran et al(28) | 205/245 | Metformin response | Dominant model | 0.65 (0.29-1.44) | 0.289 | Random | 0.025 | 72.9 |
|  |  | Phani et al(31) | 205/245 | Metformin response | Recessive model | 0.56 (0.24-1.42) | 0.230 | Random | 0.126 | 51.7 |
|  |  | Abrahams-October(33) | 205/245 | Metformin response | Additive model | 1.44 (0.81-2.56) | 0.191 | Random | 0.070 | 62.3 |

A, reference allele; a, alternative allele; OR, odd ratio; HbA1c%, glycated hemoglobin level; ΔHbA1c%, change in glycated hemoglobin level; ΔFPG, change in fasting plasma glucose level; SMD, standardized mean difference ;95% CI, 95% confidence interval; *P*^Q^ value for Q test; *P*^Z^ value for Z test;
